# Practice and Predictors of Complementary and Alternative Treatment Use Among Women With Uterine Fibroids in Lagos, Nigeria: A Cross-Sectional Study

**DOI:** 10.7759/cureus.93531

**Published:** 2025-09-30

**Authors:** Margaret A Reuben, Babatunde A Akodu, Tersur T Saalu, Oluwaseun E Familusi, Omobolanle Adenmosun, Lucky E Tietie, Chidiebere A Agbo, Charity O Maduagu, Okechukwu U Ofoegbu, Ochuwa A Babah

**Affiliations:** 1 Obstetrics and Gynecology, Lagos University Teaching Hospital, Lagos, NGA; 2 Community Health and Primary Care, College of Medicine, University of Lagos, Lagos, NGA; 3 Family Medicine, Lagos University Teaching Hospital, Lagos, NGA; 4 Obstetrics and Gynecology, College of Medicine, University of Lagos, Lagos, NGA; 5 Centre for Clinical Trials, Research and Implementation Science, College of Medicine, University of Lagos, Lagos, NGA

**Keywords:** alternative treatment, complementary treatment, practice, predictors, uterine fibroids

## Abstract

Background: Uterine fibroids are the most common benign tumors of the smooth muscles of the uterus, which can cause infertility in women of reproductive age and adverse outcomes in pregnancy, and affect the quality of life. The management of uterine fibroids includes expectant, medical, and surgical management options. Many seek alternative means of treatment because of the fear of the possible complications of surgery for uterine fibroids. This is sometimes influenced by sociocultural values and belief systems, financial constraints, or proximity to orthodox health facilities.

Objectives: The study aimed to determine the practice and predictors of use of complementary and alternative treatment among women with uterine fibroids in Lagos, Nigeria.

Methodology: This study was a cross-sectional analysis of 362 women with uterine fibroids enrolled from four publicly owned health facilities in Lagos State. Data were collected using a pretested interviewer-administered questionnaire. The questions asked comprised information on their practice and potential predictors of use of complementary and alternative treatment before seeking orthodox healthcare for uterine fibroids. Data were analyzed using the Statistical Package for the Social Sciences (SPSS) version 25 software (IBM Corp., Armonk, NY). Binary logistic regression analysis was done to identify predictors of use of complementary and alternative treatments by Nigerian women with uterine fibroids.

Results: The mean age of the respondents was 37.5 ± 6.7 years, with 282 (78.1%) married, 244 (67.4%) attained tertiary education, and 339 (93.6%) were employed. Of the women, 205 (56.6%) had used complementary and alternative methods for fibroid treatment, mostly as oral herbal concoctions 161/205 (78.5%) and vaginal inserts 21/203 (10.3%); 82/203 (40.4%) self-reported having had side effects with the use of complementary and/or alternative treatment: mostly abdominal pain 31/82 (37.8%), fever 15/82 (18.5%), and weight loss 12/82 (14.6%). Only 92/196 (46.9%) of the users found complementary and alternative methods cheaper than orthodox care. Significantly greater satisfaction was reported for orthodox care compared to complementary and alternative care (p < 0.001). Older women and those of lower parity are more likely to seek complementary and alternative care.

Conclusion: The use of complementary and alternative treatment methods for uterine fibroids is common among women presenting to the gynecological clinics for the treatment of the same condition. There is a need for targeted and focused health education intervention programs for high-risk women who may delay orthodox care through seeking complementary and alternative care for uterine fibroid treatment.

## Introduction

Uterine fibroids are the most common benign tumors of the smooth muscles of the uterus, which can be associated with infertility in women of reproductive age and adverse outcomes in pregnancy, and affect the quality of life [1,2]. Uterine fibroids are the most common tumors affecting women of reproductive age. The true incidence and prevalence of fibroids, as well as their global impact on women's health, and the role of apparent risk factors are currently unknown [3]. The burden of the condition was highlighted by the findings of the Global Burden of Disease Study, which found a 67% increase in the number of incident cases of uterine fibroids from 5.77 million in 1990 to 9.64 million in 2019 [4]. According to a US study, the incidence of uterine fibroids was 60% by the age of 35 among African-American women, with an increase to more than 80% by age 50. In comparison, Caucasian women showed a rate of 40% by the age of 35, increasing to 70% by age 50 [5].

The incidence of uterine fibroids is dependent on age and race. However, the frequency of the condition is likely to be underestimated because many women are asymptomatic or symptoms could be insidious and therefore remain undiagnosed [3,6]. Uterine fibroids are more prevalent in women of African descent, three to nine times more than Caucasians, with a peak incidence occurring at 5-10 years earlier in comparison to Caucasians [6,7].

Uterine fibroids constitute a major public health problem and the most common benign gynecological neoplasm with associated considerable morbidity affecting women of reproductive age [8]. The size and location of fibroids determine the occurrence of symptoms, the need for treatment, and the treatment options [9]. The management of uterine fibroids includes expectant, medical, and surgical management options [10].

Expectant management involves clinical observation without any intervention, as a great proportion of women diagnosed with fibroids are either asymptomatic or have minimal symptoms. Medical therapy with the use of nonhormonal and hormonal-based treatment options such as nonsteroidal anti-inflammatory drugs, antifibrinolytics, oral contraceptives, levonorgestrel-containing intrauterine systems, and gonadotropin-releasing hormone analogues is possible [10,11]. Surgical interventions still remain the mainstay of treatment for uterine fibroids, with hysterectomy, abdominal (laparotomy or laparoscopic) myomectomy, and hysteroscopic myomectomy being the most widely used [12]. Current management strategies include primarily surgical options, but the choice of treatment is guided by the patient's age and the desire to preserve fertility or avoid definitive surgical management, which is hysterectomy [5].

Presently, there is no effective long-term medical therapy for this common crippling disease [13]. A lot of research is needed in the study of uterine fibroids, as one of the major issues is that they are understudied [5]. With a great number of patients demanding nonsurgical interventions for the symptoms of fibroids, there is a growing market for selective estrogen and especially progesterone receptor modulators, and exploring the role of aromatase inhibitors, vitamin D, and green tea extract, which contains substances that are anti-inflammatory and may reduce the likelihood of developing fibroids [14].

Complementary and alternative medicine (CAM) is commonly used interchangeably to describe medical treatment, practices, and products that are not usually seen as a part of orthodox Western medicine [15]. It is broadly defined as systems of medicine that depart from conventional care and are external to the politically main health system practices [16]. Alternative care focuses on stimulating the body's ability to heal itself through energy alignment, herbal supplementation, and other balancing techniques [17].

CAM involves the use of nonconventional methods in healthcare such as herbal medicine, acupuncture, hypnosis, music, and prayer [18]. Specific methods of preventing uterine fibroids are currently not available, as the origin of the development of this condition is multifactorial. Several recent efforts to create a cheap, safe, and effective drug for uterine fibroids' prophylaxis and treatment are still in the early stages of the process. Also, recent findings suggest that substances contained in green tea, epigallocatechin gallate, vitamin D, and others, may become future preparations for chronic treatment with minimal or moderate side effects [9].

Globally, approximately 80% of people use one form of herbal medicine or the other. The reasons are the perception of the natural properties of herbal medicine, little or no side effects, delay at the hospital, ineffective orthodox medicine, control over therapy decisions, easy accessibility and availability, and lack of faith in conventional healthcare [18]. With symptomatic uterine fibroids being the most common indication for hysterectomy around the world, many women try to find uterus-preserving surgeries, nonsurgical therapies, and CAM such as dietary recommendations, medicinal herbs, homeopathy, and acupuncture [19]. Many seek alternative means of treatment as a result of fear of the possible complications of surgery for uterine fibroids, and this is sometimes influenced by the sociocultural values and belief systems, as well as financial constraints and proximity to orthodox health care facilities [1,6]. This study determined the prevalence and practice of CAM use and explored the practice and predictors of its use among women with uterine fibroids in Lagos, Nigeria. It also compared the satisfaction derived from orthodox vs. CAM for the treatment of uterine fibroids.

## Materials and methods

Study design

The study was a descriptive cross-sectional study. The study was conducted from February 7, 2023, to May 29, 2023.

Study location

The study was conducted at one tertiary healthcare facility, Lagos University Teaching Hospital (LUTH), Idi-Araba, Lagos, and three secondary healthcare facilities within Lagos State, which were purposefully selected due to their proximity to one another, facilitating the coordination of the research. The secondary healthcare facilities are Maternal and Child Centre, Amuwo-Odofin; Mother and Child Centre, Gbaja, Surulere; and Lagos Island Maternity Hospital (LIMH), Lagos Island.

Study population

The study was conducted among women diagnosed as having uterine fibroids on pelvic ultrasonography at the various health facilities serving as study sites within Lagos, Nigeria. Included in the study were women already diagnosed with uterine fibroids who were registered at the gynecological outpatients' clinics of the study sites and gave informed consent. Excluded from this study were all women with a diagnosis other than uterine fibroid and women in whom a diagnosis had not been made.

Sample size determination

Considering the prevalence of uterine fibroids in Lagos, Nigeria, to be 31% as found in an earlier study by Oluwole et al. [20], and using Cochran's formula, a sample size of 362 was considered adequate to power this study at a 95% confidence level, considering a 10% attrition rate.

Sampling technique

To select the health facilities, a list of public secondary health facilities in Lagos State was obtained from the Nigeria Health Facility Registry [21]. Three secondary health facilities were purposively selected due to their proximity to one another. A tertiary health facility (LUTH) was also purposively selected because it is the author's training institution.

To select the study participants, the total sample from each of the selected health facilities was the expected total number of fibroid cases seen at the Gynecology clinic of each facility over the study period. The duration of the participant enrolment for this study was three months. The total number of gynecological patients seen quarterly was obtained from the medical records of each health facility, with about 629 gynecological patients seen quarterly in Maternal and Child Centre, Amuwo-Odofin, 507 in Mother and Child Centre, Gbaja, Surulere, 3,203 in LIMH, and 1,154 in LUTH as seen in the medical records of the previous year, 2022. Considering 31% of gynecological patients as the prevalence of women with uterine fibroids as found in a previous study [20], this was used in the calculation of the estimated number of women with uterine fibroids presenting at the various clinics; 195 in Maternal and Child Centre, Amuwo-Odofin, 157 in Mother and Child Centre, Gbaja, Surulere, 993 in LIMH, and 358 in LUTH as calculated using the medical records of the previous year, 2022.

Using proportionate sampling, the number of participants enrolled per study site was calculated as the estimated total number of women with fibroids per site divided by the estimated total number of women with fibroids in all study sites. A systematic sampling technique was used to select the study participants. The case notes of all women with uterine fibroids presenting on each clinic day at each study site were pooled together to make a selection. Where a woman had previously participated, her case note was excluded.

Sampling interval estimation

A sampling interval of 5 was calculated by dividing the estimated total number of women with uterine fibroids in all study sites in a quarter (1,703) by the sample size (362) for this study. The starting point for participant selection was determined at each site on a range between 1 and the sample interval of 5, which was determined by simple random sampling. The sampling interval was applied to systematically select subsequent participants (which was every fifth woman with uterine fibroids) until the required sampling size for each site was achieved.

Data collection tools and techniques

A standard questionnaire was used for data collection, and it was adapted from the studies done on the “Health seeking behavior of Tiv women living with fibroid in Benue State, Nigeria”; and “Knowledge of, perception of, and attitude toward uterine fibroids among women with fibroids in Lagos, Nigeria” respectively, keeping in mind the objectives of this study [1,6].

The questionnaires were interviewer-administered. The questionnaire contained both open-ended and closed-ended questions with an option of free response, when such a response was not in the checklist provided. It consisted of a total of 40 questions. The questions were asked in four sections, labeled sections A-D.

Section A dealt with the sociodemographic characteristics of the respondent. It consisted of 14 questions. Section B dealt with the practices used as complementary and alternative treatment before seeking orthodox healthcare for uterine fibroids. It consisted of 15 questions. Section C dealt with the factors influencing the use of complementary and alternative treatment for uterine fibroids. It consisted of two questions. Section D dealt with the levels of satisfaction on the use of complementary and alternative treatment vs. orthodox healthcare in the management of uterine fibroids. It consisted of nine questions (see the Appendix).

Pretesting

A pretest of the data collection tool was conducted to help detect ambiguous words, misinterpretation of questions, and flow of questions, to estimate the length of time to complete the survey, and to determine the relevance of the questionnaire to the study objectives. Pretesting was done on 10 women in the gynecological outpatient clinic in LUTH. The internal consistency of the questionnaire was found to be 0.7646 using the scale reliability coefficient calculated on Cronbach's alpha. This was considered satisfactory because it was greater than 0.7. The questionnaire was slightly modified based on findings from pretesting. Data obtained from pretesting were not included in the final data that were analyzed.

Data analysis

Data quality checks for completeness and inconsistencies were done after the data collection. Microsoft Excel (Microsoft Corporation, Redmond, WA) was used for data entry, while Statistical Package for the Social Sciences version 25 software (IBM Corp., Armonk, NY) was used for analysis. Descriptive statistics were done, while continuous variables were presented as mean and standard deviation. Categorical variables were presented as frequency and percentage. The prevalence of use of complementary and alternative treatment for uterine fibroids was presented as a percentage. Chi-square test was used to test for the association between sociodemographic variables and the decision to use CAM. Binary logistic regression analysis was used to identify determinants for the use of alternative methods for the treatment of uterine fibroids. The level of satisfaction was recategorized as unsatisfied (0-3), moderately satisfied (4-6), and satisfied (7-10). A subgroup analysis of women who had alternative treatment previously and now sought orthodox care was carried out. Their median satisfaction score for both treatment methods was presented as proportions and compared using the Wilcoxon signed-rank test after testing for normality with the Kolmogorov-Smirnov test. The level of satisfaction for use of both treatment methods (CAM vs. orthodox) by the participants was compared using Fisher's exact test. There was no imputation for missing values. Statistical significance was set at p <0.05 using a two-tailed hypothesis.

Ethical considerations

Ethical approval was obtained from the Health Research and Ethics Committee of LUTH (approval number: ADM/DSCST/HREC/APP/5553). Administrative approval to use the secondary healthcare facilities was obtained from Lagos State Health Service Commission (approval number: LSHSC/2222/VOLV/12). Written informed consent was obtained from each respondent before the questionnaires were administered. Confidentiality of information obtained was assured. Respondents were not asked to write identifying information such as their names, phone numbers, or addresses on the questionnaires.

## Results

Three hundred and sixty-two questionnaires were administered altogether in the four study sites. The mean ± SD age of the respondents was 37.5 ± 6.7 years. Most of the women recruited in this study, 184 (51.0%), were between the age group of 35 and 44 years. A predominant number of women, 282 (78.1%), were married, had a tertiary level of education, 244 (67.4%), and were employed, 339 (93.6%). Table 1 shows the details of the sociodemographic characteristics of the respondents.

**Table 1:** Sociodemographic characteristics of respondents (n = 362)

Variable	Frequency	Percentage (%)
Age (years), n = 361		
15-24	3	0.8
25-34	119	33.0
35-44	184	51.0
≥45	55	15.2
Marital status, n = 361		
Single	66	18.3
Married	282	78.1
Separated/divorced	9	2.5
Widowed	4	1.1
Ethnicity, n = 362		
Yoruba	172	47.5
Hausa	8	2.2
Igbo	128	35.4
Others	54	14.9
Religion, n = 362		
Christianity	262	72.4
Islam	100	27.6
Level of education, n = 362		
No formal education	2	0.5
Primary	10	2.8
Secondary	106	29.3
Tertiary	244	67.4
Employment status, n = 362		
Employed	339	93.6
Unemployed	23	6.4
Monthly salary, n = 362		
None	23	6.4
Less than ₦30,000	24	6.6
₦30,000 to ₦50,000	60	16.6
Greater than ₦50,000 to ₦100,000	109	30.1
Greater than ₦100,000	129	35.6
Did not disclose salary	17	4.7

Table 2 shows the clinical profile of the women with uterine fibroids. Their median parity was 0 (interquartile range, IQR: 0-1). The onset of symptoms to diagnosis was within one year for only 30% of the women; 12 (3.5%) having been diagnosed less than one month, 31 (8.9%) diagnosed between one and six months, and 61 (17.6%) diagnosed between greater than six and twelve months, with most cases 145 (41.8%) diagnosed between one and five years.

**Table 2:** Clinical profile in women with uterine fibroids (n = 362) IQR: interquartile range

Clinical characteristic	Value
Age at menarche, median (IQR), n = 340	14 (13-16)
Parity, median (IQR), n = 353	0 (0-1)
Onset of symptoms to diagnosis of uterine fibroid, n (%), n =347	
Less than 1 month	12 (3.5)
1-6 months	31 (8.9)
Greater than 6 months to 12 months	61 (17.6)
Greater than 1 year to 5 years	145 (41.8)
Greater than 5 years to 10 years	72 (20.7)
Greater than 10 years	26 (7.5)
Clinical presentations of uterine fibroid, n (%), n = 362	
Heavy menstrual bleeding	187 (51.7)
Abdominal mass/swelling	109 (30.1)
Inability to conceive	100 (27.6)
Irregular/prolonged menstruation	77 (21.3)
Abdominal pain	51 (14.1)
Urinary symptoms (frequency, urgency)	41 (11.3)
Dysmenorrhea	9 (2.5)
Dizziness	4 (1.1)
Bleeding in pregnancy	3 (0.8)
Dyspareunia	2 (0.6)
Weight loss	1 (0.3)
Method of diagnosis, n (%), n = 359	
Pelvic ultrasonography	240 (66.9)
Clinical examination	80 (22.3)
Histology	26 (7.2)
Surgery	12 (3.3)
Computerized tomography scan	1 (0.3)

The commonest symptoms were heavy menstrual bleeding in 187 (51.7%) of the women, abdominal mass/swelling in 109 (30.1%), inability to conceive in 100 (27.6%), irregular menstruation in 77 (21.3%), and abdominal pain in 51 (14.1%). The majority of cases were diagnosed with an ultrasound scan, 240 (66.9%), and clinical examination, 80 (22.3%).

According to this study, 205 (56.6%) of women sought alternative care first for the management of uterine fibroids before presenting for orthodox treatment. Table 3 shows details of the practices of alternative care by women with uterine fibroids who self-reported having sought CAM previously. The CAM received were mostly herbal concoctions taken by mouth, 161 (78.5%), nutritional supplements, 43 (21.2%), and insertion of herbal preparations into the vagina, 21 (10.3%).

**Table 3:** Practices of complementary and alternative care by respondents prior to seeking orthodox healthcare (n = 205) ^*^Reign soap refers to an herbal-branded bath soap used for the treatment of fibroids and their symptoms. Prophetic medicine refers to herbal mixtures from spiritual centers and churches used for the treatment of fibroids and their symptoms, administered through multiple routes. None of the participants used hypnosis as a complementary and alternative treatment

Variable	Frequency	Percentage (%)
Types of complementary and alternative treatment^*^		
Herbal concoction taken by mouth, n =205	161	78.5
Herbal medicine inserted into the vagina, n = 203	21	10.3
Nutritional supplements, n = 203	43	21.2
Acupuncture, n = 203	3	1.5
Music, n = 203	1	0.5
Prayer, n = 203	20	9.9
Prophetic medicine, n = 203	1	0.5
Reign soap, n = 203	1	0.5
Anal substance/inserts, n = 203	1	0.5
Frequency of usage, n = 205		
Just once	2	1.0
Once a day	42	20.5
Twice a day	89	43.4
Three times a day	38	18.5
More than 3 times a day	16	7.8
Once a week	14	6.8
Twice a week	1	0.5
Once a month	3	1.5
Duration of use, n = 196		
Less than 3 months	50	25.5
3-6 months	42	21.4
>6-12 months	57	29.1
>1-5 years	42	21.4
>5-10 years	4	2.1
Greater than 10 years	1	0.5

The substances used for treatment were mostly used at daily frequencies: once a day by 42 (20.5%), twice daily by 89 (43.4%), and three times a day by 38 (18.5%) of the women. Most of the women had used the preparations for less than five years; 50 (25.5%) for less than three months, 42 (21.4%) for three to six months, 57 (29.1%) for six to twelve months, and 42 (21.4%) for one to five years.

Tables 4, 5 show details of the respondents' perceived effectiveness and self-reported incidence of side effects for the various CAMs used. Only about a quarter, 50 (26.6%), considered the CAM they used to be effective. The measure of effectiveness was considered in the form of reduced heavy menstrual bleeding in 20 (40.0%) of these women, a reduction in the size of the abdominal mass/swelling in 14 (28.0%), and a reduction in the severity of abdominal pain in 12 (24.0%). Almost half of the respondents who have had CAM found it to be cheaper than orthodox care, 92 (46.9%). However, 55 (28.1%) considered it to be similar in cost to orthodox care, while 44 (22.4%) found it more expensive than orthodox treatment.

**Table 4:** Perceived effectiveness of alternative and complementary methods use among the respondents (n = 205)

Variable	Frequency	Percentage (%)
Effectiveness of choice complementary and alternative treatment, n = 188		
Effective	50	26.6
Indifferent	62	33.0
Ineffective	76	40.4
Areas of improvement if any, n = 50		
Reduced heavy menstrual bleeding	20	40.0
Reduced abdominal mass/swelling	14	28.0
Able to conceive	5	10.0
Reduced abdominal pain	12	24.0
Reduced painful sex	1	2.0
Reduced body weight	1	2.0
Comparison of complementary and alternative treatment to orthodox healthcare, n = 196		
Cheaper	92	46.9
Similar in cost	55	28.1
More expensive	44	22.4
I don’t know	5	2.6

**Table 5:** Self-reported incidence of side effects of alternative and complementary methods used by the respondents (n = 205)

Variable	Frequency	Percentage (%)
Self-reported incidence of side effects of complementary and alternative treatment, n = 203		
Yes	82	40.4
No	121	59.6
Side effects of complementary and alternative treatment (n = 82)		
Abdominal pain/discomfort	31	37.8
Bleeding per vagina	4	4.9
Vaginal tightening	1	1.2
Painful urination	2	2.4
Fever	15	18.3
Inability to have sexual intercourse	1	1.2
Infection	6	7.3
Diarrhea	11	13.4
Dehydration	1	1.2
Weakness	5	6.1
Weight loss	12	14.6
Increased abdominal mass/swelling	1	1.2
Skin changes	3	3.7
Vaginal discharge	1	1.2
Forms of complementary and alternative treatment that caused side effects, n = 65		
Acupuncture	3	4.6
Alcohol-based concoction	14	21.5
Meal planning	1	1.5
Nutritional supplements	9	13.8
Water-based concoction	30	46.2
Vaginal inserts (grinded leaves, liquid mixture)	8	12.3

Regarding self-reports on the side effects, 82 (40.4%) of those who had CAM experienced at least a side effect, mostly in the form of abdominal pain or discomfort in 31 (37.8%), fever in 15 (18.3%), weight loss in 12 (14.6%), and diarrhea in 11 (13.4%). The form of CAM that was perceived to have caused the side effects was a water-based concoction in 30 (46.2%) of the women and an alcohol-based concoction in 14 (21.5%).

Table 6 shows the association between sociodemographic factors and the decision to seek CAM among the respondents. Of the sociodemographic factors, only age was found to be significantly associated with the decision to seek CAM. More women in the younger age group, 20-30 years, did not use CAM before orthodox care, 44 (28.2%), compared to women in the same age group who sought CAM, 20 (9.8%), whereas a significantly greater proportion of women in the older age group above 30 years used CAM (p < 0.001).

**Table 6:** Association between sociodemographic factors and decision to seek alternative care for fibroid management (n = 362) ₦: Nigerian Naira. During the study period, February to May 2023, $1 was equivalent to ₦460.42. Chi-square test used for hypothesis testing

Variable	Used alternative or complementary method (n = 205), n (%)	Did not use alternative or complementary method (n = 157), n (%)	Chi-square test statistics	p value
Age (years), n = 361				
20-30	20 (9.8)	44 (28.2)	22.056	<0.001
31-40	111 (54.1)	75 (48.1)		
>40	74 (36.1)	37 (23.7)		
Marital status, n = 361				
Single/separated/divorced/widowed	40 (19.6)	39 (24.8)	2.190	0.335
Married	164 (80.4)	118 (75.2)		
Ethnicity, n = 362				
Yoruba	90 (43.9)	82 (52.2)	3.480	0.323
Hausa	6 (2.9)	2 (1.3)		
Igbo	75 (36.6)	53 (33.8)		
Others	34 (16.6)	20 (12.7)		
Religion, n = 362				
Christianity	152 (74.1)	110 (70.1)	0.741	0.389
Islam	53 (25.9)	47 (29.9)		
Level of education, n = 362				
Secondary	60 (29.3)	58 (36.9)	2.383	0.123
Tertiary	145 (70.7)	99 (63.1)		
Salary, n = 345				
Less than ₦30,000 or none	26 (13.3)	21 (14.1)	1.133	0.568
₦30,000 to less than ₦100,000	92 (46.9)	77 (51.7)		
₦100,000 and above	78 (39.8)	51 (34.2)		
Parity, n = 353				
0	128 (64.0)	92 (60.1)	3.438	0.179
1	34 (17.0)	20 (13.1)		
≥2	38 (19.0)	41 (26.8)		

Table 7 shows findings from binary logistic regression analysis identifying predictors of the decision to seek CAM. Only age and parity were identified as significant predictors of the decision to seek care among women with uterine fibroids. Compared to women aged 40 years and above, younger women aged 20-30 had 87% lower odds of seeking CAM (adjusted odds ratio, aOR: 0.13; 95% confidence interval, CI: 0.06-0.30), and women aged 31-40 had 49% lower odds of seeking CAM (aOR: 0.51; 95% CI: 0.29-0.91). This suggests a direct association between a woman's age and the odds of her seeking CAM; the older a woman with uterine fibroids, the more likely it is that she would want to seek treatment with CAM.

**Table 7:** Factors influencing decision to seek alternative care for management of uterine fibroid among respondents (n = 362) ₦: Nigerian Naira. During the study period, February to May 2023, $1 was equivalent to ₦460.42. Multivariable logistic regression analysis was conducted in six steps; retained in the final model were age and parity. Goodness of fit for the multivariable model using Hosmer and Lemeshow p value is 0.902 CI: confidence interval; OR: odds ratio

Predictor	Univariable analysis		Multivariable analysis	
	OR (95% CI)	p value	OR (95% CI)	p value
Age (years)				
20-30	0.23 (0.12-0.44)	<0.001	0.13 (0.06-0.30)	<0.001
31-40	0.74 (0.45-1.21)	0.230	0.51 (0.29-0.91)	0.021
>40	Ref	-	Ref	-
Marital status				
Single/separated/divorced/widowed	0.74 (0.45-1.22)	0.234	1.05 (0.57-1.94)	0.879
Married	Ref	-	Ref	-
Ethnicity				
Yoruba	Ref	-	Ref	-
Hausa	2.73 (0.54-13.92)	0.226	3.32 (0.56-19.74)	0.187
Igbo	1.29 (0.81-2.05)	0.281	1.47 (0.79-2.71)	0.221
Others	1.55 (0.83-2.90)	0.172	1.57 (0.72-3.44)	0.260
Religion				
Christianity	1.24 (0.77-1.95)	0.390	0.72 (0.37-1.37)	0.316
Islam	Ref	-	Ref	-
Level of education				
Secondary	0.71 (0.45-1.10)	0.123	0.65 (0.39-1.10)	0.106
Tertiary	Ref	-	Ref	-
Salary				
Less than ₦30,000 or none	0.81 (0.41-1.59)	0.539	1.00 (0.47-2.10)	0.991
₦30,000 to less than ₦100,000	0.78 (0.49-1.24)	0.298	0.88 (0.52-1.50)	0.641
Greater than or equal to ₦100,000	Ref	-	Ref	-
Parity				
0	1.50 (0.90-2.52)	0.123	2.78 (1.50-5.14)	0.001
1	1.83 (0.90-3.72)	0.093	2.22 (1.05-4.70)	0.036
≥2	Ref	-	Ref	-

On the other hand, parity has an inverse association with the odds of a woman with uterine fibroids seeking CAM. In comparison to multiparous women (para 2 and above), less parous women had significantly higher odds of seeking CAM. Nulliparous women (para 0) had almost three times greater odds of seeking CAM (aOR: 2.78; 95% CI: 1.50-5.14), and primiparous women (para 1) had greater odds of seeking CAM for fibroid treatment (aOR: 2.22; 95% CI: 1.05-4.70).

Other factors like marital status, ethnicity, religion, educational attainment, and income were not found to be associated with the odds of a woman with uterine fibroids seeking treatment with CAM.

Table 8 shows the comparison of satisfaction with both methods among the subgroup of women who have had both CAM and orthodox care at some time. The median score for level of satisfaction was significantly higher with orthodox care, 7 (IQR: 6-9), compared to alternative care received, 3 (IQR: 1-5) (p < 0.001). Significantly more women with uterine fibroids reported being satisfied with orthodox care compared to CAM (p = 0.016).

**Table 8:** Comparison of alternate/complementary treatment versus orthodox treatment among women with uterine fibroids who have used both methods (n = 200) ^*^Wilcoxon rank-sum test; ^#^Fisher's exact test; Z-statistics value Missingness: 5 nonresponses for satisfaction with both methods IQR: interquartile range

Parameter	Complementary/alternative method	Orthodox method	Test statistics	p value
Satisfaction score, median (IQR)	3 (1-5)	7 (6-9)	-11.307^¤^	<0.001^*^
Level of satisfaction, n (%)				
Unsatisfied	124 (62.0)	5 (2.5)	-	0.016^#^
Moderately satisfied	53 (26.5)	69 (34.5)		
Satisfied	23 (11.5)	126 (63.0)		

## Discussion

This study examined the practice and predictors of use of CAM among women with uterine fibroids. We found that over half of the women with uterine fibroids used CAM before seeking orthodox care, mostly in the form of oral herbal concoctions in almost four in five of the women and as vaginal inserts in one in 10 of them. Among women who had used both methods, two in five experienced side effects with CAM, which included abdominal discomfort, fever, and weight loss. Women less than 40 years old are less likely to seek CAM for fibroid treatment, whereas women of low parity have twice the odds of seeking CAM compared to the multiparous women.

In our sample, there was a larger proportion of women in the older age group of above 35 years. This age prevalence of women with uterine fibroids is similar to the cross-sectional study done in Ghana, in which fibroids were found to be prevalent mostly in women above 35 years [8]. A systematic review also showed a four- to ten-fold likelihood of uterine leiomyomata in women above 40 years [3].

Our finding that older women above 35 years are more likely to use CAM for the treatment of uterine fibroids is unique, as earlier studies did not explore the association between age and the use of CAM. However, studies have corroborated our findings that a large proportion of women in our clinics used CAM for the treatment of fibroids before presenting for orthodox treatment [6,22]. Similar to this study was the finding that a lot of women sought care in spiritual homes (churches, mosques), traditional healers, and massage parlors, thereby presenting late to the hospital [6]. The parity of women with uterine fibroids in this study aligns with earlier reports that nulliparity is a risk factor for the development of uterine fibroids [3,6,22].

Heavy menstrual bleeding, abdominal mass/swelling, inability to conceive, irregular menstruation, and abdominal pain were found to be the commonest presentations of uterine fibroid in this study. Uterine fibroids may be associated with infertility and are an infrequent primary cause of infertility [6,23]. Heavy, irregular menstrual bleeding and abdominal pain can have a negative impact on women's lives and require intervention [24]. Most cases of uterine fibroids were diagnosed with ultrasound and clinical examination in this study. An ultrasound is the gold standard test for uterine fibroids, as it is generally available and enables easy and cheap confirmation in almost all instances, while a pelvic examination may reveal an enlarged uterus or mass [5].

Another interesting finding is that only a quarter of the women in our sample who used CAM used the method for a short duration of less than three months, suggesting that most women spend more time using these methods without seeking orthodox care. This delay in seeking treatment may partly explain why women present to gynecology clinics with huge uterine fibroids that have advanced over time, making surgical interventions more challenging.

The association of age and parity as independent determinants of the use of CAM for uterine fibroids, with no association observed with sociodemographic variables like education, religion, marital status, ethnicity, and income, is corroborated by an earlier report from a survey in Nigeria by WHO, which revealed that about 75% of the population patronized traditional medicine irrespective of the social, educational, or religious background of the people, which is indicative of acceptance of herbal medical practice. CAM is regarded as less expensive than orthodox medicine [25]. Previously, a study emphasized that the high cost of herbal products deterred people from using herbal medicine [18]. The latter might not be the case because about half of the women in our sample considered CAM for fibroid treatment to be expensive or at least similar in cost to orthodox care. There is a need for greater exploration into the facilitators and barriers to seeking orthodox care for uterine fibroids through the conduct of qualitative research.

The CAM used was mostly a herbal concoction taken by mouth. CAM was found in this study to be effective in about 26.6% of respondents, with the measure of effectiveness experienced as reduced heavy menstrual bleeding, size of abdominal mass/swelling, and severity of abdominal pain. There is a potential for toxicity with the use of CAM because often there is no recommended dosage, and they are consumed in large amounts and/or frequently; their chemical compositions are unknown, some might contain impurities or interact with other orthodox medications the woman may be taking for other conditions. This is why the use is usually not encouraged in routine gynecological practice in Nigeria. Those who often prescribe CAM in the country are unskilled persons. To determine the clinical efficacy and safety of herbal preparations in the treatment of uterine fibroids, large-scale clinical studies are needed [6].

The study showed that a considerably greater proportion of women with uterine fibroids reported higher levels of satisfaction with orthodox care compared to CAM. The women who are more likely to seek treatment for uterine fibroids with CAM are those with low parity, and this is the group of women who are likely to have infertility complicating their condition. This reiterates the need for focused health education intervention programs geared toward the high-risk women in the older age group and low parity who have uterine fibroids and who are more likely to seek CAM. It is worth evaluating in future research the proportion of women with uterine fibroids and background infertility who conceived after treatment with the orthodox method, as the findings of such a study could strengthen the content of counseling for women with uterine fibroids. It is also important to improve access to reproductive healthcare for women with uterine fibroids.

Strengths and limitations

This study used a mix of secondary and tertiary healthcare facilities in Lagos as study sites to capture women with diverse characteristics. However, sampling of healthcare facilities was nonprobabilistic with a risk of bias. The study was able to clearly bring to light the association between age and orthodox care and helped to identify those women who are likely to seek CAM. A vital limitation is that the study is hospital-based, and the findings may not be representative of the general population of women with uterine fibroids. Furthermore, the data were self-reported responses, and this is prone to recall bias. In addition, there is a risk of social desirability bias in this study because some respondents can overreport desirable perceptions and attitudes and underreport those that are undesirable. However, it is our belief that the findings of this study would improve scientific knowledge and serve as a basis for focused health education intervention programs for women living with uterine fibroids so that they can make better informed decisions regarding the type of care to seek for their condition.

## Conclusions

The use of CAM for uterine fibroids is common among women presenting to the gynecological clinics for treatment for the same condition in Nigeria. The older age group and women of lower parity are more likely to seek CAM and should be considered an important target population during focused health education intervention programs on uterine fibroids. Self-reported safety issues reported by the respondents in this study in the form of adverse events corroborate the need to discourage the use of CAM for uterine fibroid treatment. The need for the latter is further strengthened by the greater satisfaction associated with orthodox treatment. This underscores the need for health education of women, not only within the health facility but also within the community.

## Appendices

Section A: sociodemographic data

Please fill/tick as appropriate

1. How old are you as of your last birthday (in years)? ________

2. What is your marital status?

Single (  )      Married (  )        Separated/Divorced (  )      Widowed (  )

3. What tribe/ethnic group do you belong to?

Yoruba (  )       Hausa (  )         Igbo  (  )    Others, please specify:__________

4. What is your religion?

Christianity (  )       Islam (  )           Others, please specify: __________

5. What is the highest level of education you have completed?

Primary (  )          Secondary (  )           Tertiary (  )           Postgraduate (  )

No formal education (  )

6. What is your occupation? _______________________________________

7. What is the highest level of education your spouse/partner has completed?

Primary (  )         Secondary (  )           Tertiary (  )            Postgraduate (  )

No formal education (  )

8. What is your spouse/partner’s occupation? ___________________________________

9. What is your monthly income from all sources?

None (  )     <30,000 (  )      30,000-50,000 (  )      51,000-100,000 (  )      101,000-200,000 (  )   201,000-300,000 ( )         >300,000 (  )

10. At what age did you have your first menstruation (menarche)? _______

11. Have you ever been pregnant at all? Yes (  )               No (  )

If yes to Q11, please respond to the following Q12, Q13, and Q14.

12. At what age were you delivered of your first child (in years)? _______

13. At what age were you delivered of your last child (in years)? _______

14. How many pregnancies have you carried for up to 6 and a half months (28 weeks)?

None (  )        One (  )        Two (  )       Three (  )        Others, please specify: __________

Section B: practices used as complementary and alternative treatment prior to seeking orthodox healthcare for uterine fibroids

15. What do you understand by uterine fibroids? _____________________________________________________________________

_____________________________________________________________________

_____________________________________________________________________

_____________________________________________________________________

16. How was the diagnosis that you have/had uterine fibroids made? __________

Pelvic USS (  )          Clinical examination (  )        Surgery (  )           Histology (  )

Others, please specify: __________

17. How long have you had uterine fibroids (specify in months or years)? __________

18. What complaints did you have about the presentation on uterine fibroids?

Heavy menstrual bleeding (  )

Irregular/prolonged menstruation  (  )

Abdominal mass/swelling            (  )

Urinary symptoms (frequency, urgency)         (  )

Inability to conceive       (  )

Others, please  specify:_________

19. What does complementary and alternative treatment mean to you? (You may choose more than one answer)

Treatment methods that are not within the usual medical or hospital-based approach to care (  )

Herbal medicine            (  )

Nutritional/dietary supplements         (  )

Acupuncture        (  )

Hypnosis          (  )

Music         (  )

Prayer        (  )

Others, please specify:_________

20. Did you use complementary and alternative treatment elsewhere before seeking orthodox healthcare in a hospital for the management of uterine fibroids? Yes (  )               No (  )

If yes to Q20, please respond to Q21 to Q29.

21. What form(s) of complementary and alternative treatment did you use before seeking orthodox healthcare for the management of uterine fibroids? (You may choose more than one answer)

Herbal concoction taken by mouth    (  )    please  specify:_________

Herbal medicine inserted into the vagina    (  )    please  specify:_________

Nutritional supplements         (  )   please  specify:_________

Acupuncture        (  )

Hypnosis          (  )

Music      (  )

Prayer      (  )

Others, please  specify:_________

22. How often do/did you use this/these form(s) of complementary and alternative treatment?

Once a day (  )          Twice a day (  )       Three times a day (  )            More than three times a day (  )           Once a week (  )          Once a month (  )          Others, please specify: _________

23. How long did you use this/these form(s) of complementary and alternative treatment?

<3 months (  )           3-6 months (  )           >6 months to 1 year (  )

Others, please specify: __________

24. How effective was/were this/these form(s) of complementary and alternative treatment?

**Table 9:** 24. How effective was/were this/these form(s) of complementary and alternative treatment?

Ineffective	Somewhat ineffective	Indifferent	Somewhat effective	Very effective
-	-	-	-	-
-	-	-	-	-
-	-	-	-	-

25. If you consider the complementary and alternative treatment effective, in which aspect did you notice improvement?

Reduced heavy menstrual bleeding     (  )

Reduced abdominal mass/swelling       (  )

Able to conceive       (  )

Others, please specify:________

26. In comparison to orthodox healthcare which you are receiving now, were this/these form(s) of complementary and alternative treatment cheaper or more expensive?

Complementary/alternative treatment is cheaper     (  )

Complementary/alternative treatment is more expensive     (  )

Complementary/alternative treatment is similar in cost to orthodox healthcare   (  )

I don’t know   (  )

27. Is there any side effect with the use of this/these form(s) of complementary/alternative treatment?

Yes (  )               No (  )

28. What were the side effects?

Abdominal pain    (  )

Bleeding per vaginam    (  )

Vagina tightening     (  )

Painful urination     (  )

Fever      (  )

Inability to have sexual intercourse (  )

Infection  (  )

Others, please specify:________

29. Which of this/these form(s) of complementary/alternative treatment caused these side effects? Please  specify:_________

Section C: factors influencing the use of complementary and alternative treatment for uterine fibroids

30. Why did you choose to use complementary/alternative treatment instead of orthodox healthcare for the management of uterine fibroids? (You may choose more than one answer)

Fibroid is best treated with herbs    (  )

Fear of surgery     (  )

Complementary/alternative care has little or no side effect     (  )

Orthodox medicine is ineffective     (  )

Easy accessibility and availability of complementary/alternative care (  )

Delay at the hospital (  )

Others, please specify:________

31. What influenced your choice of complementary/alternative treatment for uterine fibroids over orthodox healthcare?

Cost of orthodox healthcare (investigations and surgery)    (  )

Family, relatives, and friends’ pressure/influence     (  )

Spiritual/religious beliefs     (  )

Cultural beliefs     (  )

Social media      (  )

Others, please specify:________

Section D: levels of satisfaction with the use of complementary/alternative treatment vs. orthodox healthcare in the management of uterine fibroids

32. Complementary/alternative treatment is cheaper and readily available.

a.     I strongly agree     (  )

b.     I agree     (  )

c.     Uncertain     (  )

d.     I disagree      (  )

e.     I strongly disagree     (  )

33. Complementary/alternative treatment produces faster and more effective results in the management of uterine fibroids.

a.     I strongly agree    (  )

b.     I agree     (  )

c.     Uncertain     (  )

d.     I disagree     (  )

e.     I strongly disagree     (  )

34. There is little or no side effect with the use of complementary/alternative treatment.

a.     I strongly agree    (  )

b.     I agree     (  )

c.     Uncertain     (  )

d.     I disagree     (  )

e.     I strongly disagree     (  )

35. Fertility is preserved with the use of complementary/alternative treatment.

I strongly agree    (  )

I agree    (  )

Uncertain     (  )

I disagree     (  )

I strongly disagree     (  )

36. I am more confident and believe in complementary/alternative treatment than orthodox medicine.

I strongly agree    (  )

I agree     (  )

Uncertain     (  )

I disagree     (  )

I strongly disagree     (  )

37. Orthodox healthcare involves a prolonged process of care in comparison to complementary/alternative care.

I strongly agree    (  )

I agree     (  )

Uncertain     (  )

I disagree     (  )

I strongly disagree     (  )

38. Complementary/alternative care practitioners are more welcoming than orthodox healthcare personnel (doctors, nurses, etc.).

I strongly agree    (  )

I agree    (  )

Uncertain     (  )

I disagree     (  )

I strongly disagree     (  )

39. I do not want my uterus (womb) to be tampered with.

I strongly agree    (  )

I agree     (  )

Uncertain     (  )

I disagree     (  )

I strongly disagree     (  )

40. Overall satisfaction rating

On a scale of 0 to 10 (0 representing not at all satisfied, and 10 being extremely satisfied with the treatment approach), rate your level of satisfaction with both methods.

Complementary/alternative care   0       1       2       3       4       5       6       7       8       9       10

Orthodox care                                0       1       2       3       4       5       6       7       8       9       10
